# Identification of Potential Artefacts in In Vitro Measurement of Vanadium-Induced Reactive Oxygen Species (ROS) Production

**DOI:** 10.3390/ijerph192215214

**Published:** 2022-11-18

**Authors:** Iwona Zwolak, Ewa Wnuk, Michał Świeca

**Affiliations:** 1 Department of Biomedicine and Environmental Research, The John Paul II Catholic University of Lublin, Konstantynów Ave. 1J, 20-708 Lublin, Poland; 2 Department of Biochemistry and Food Chemistry, University of Life Sciences, Skromna Str. 8, 20-704 Lublin, Poland

**Keywords:** vanadium, ROS, oxidative stress, DCFH_2_-DA, DHR 123, culture media

## Abstract

We investigated vanadium, i.e., a redox-active heavy metal widely known for the generation of oxidative stress in cultured mammalian cells, to determine its ability to interfere with common oxidative stress-related bioassays in cell-free conditions. We first assessed the prooxidant abilities (H_2_O_2_ level, oxidation of DHR 123, and DCFH-DA dyes) and antioxidant capacity (ABTS, RP, OH, and DPPH methods) of popular mammalian cell culture media, i.e., Minimal Essential Medium (MEM), Dulbecco’s Minimal Essential Medium (DMEM), Dulbecco’s Minimal Essential Medium-F12 (DMEM/F12), and RPMI 1640. Out of the four media studied, DMEM has the highest prooxidant and antioxidant properties, which is associated with the highest concentration of prooxidant and antioxidant nutrients in its formulation. The studied vanadium compounds, vanadyl sulphate (VOSO_4_), or sodium metavanadate (NaVO_3_) (100, 500, and 1000 µM), either slightly increased or decreased the level of H_2_O_2_ in the studied culture media. However, these changes were in the range of a few micromoles, and they should rather not interfere with the cytotoxic effect of vanadium on cells. However, the tested vanadium compounds significantly stimulated the oxidation of DCFH-DA and DHR123 in a cell-independent manner. The type of the culture media and their pro-oxidant and antioxidant abilities did not affect the intensity of oxidation of these dyes by vanadium, whereas the vanadium compound type was important, as VOSO_4_ stimulated DCFH-DA and DHR oxidation much more potently than NaVO_3_. Such interactions of vanadium with these probes may artefactually contribute to the oxidation of these dyes by reactive oxygen species induced by vanadium in cells.

## 1. Introduction

Vanadium (V) exhibits important physical, chemical, and biological properties, which are useful in industrial applications and medicine products. For example, as a steel additive, V is highly demanded by the steel industry sector. In addition, it is also useful in the ceramics, glass, photographic, and chemical industry [1] and is increasingly being used in V redox flow batteries [2]. The biological activities of V include those with potential medicinal applications such as anticancer, antidiabetic, and antiviral effects [3]. However, V is also an environmental contaminant and a subject of concern among toxicologists [4,5,6,7]. Disorders related to human exposure to V include symptoms in the respiratory system [8,9], cardiovascular system [10], foetal growth disorders [11], and altered neurobehavioral functions [12]. Vanadium in the form of vanadium pentoxide has been classified by International Agency for Research on Cancer (IARC) as possibly carcinogenic to humans (Group 2B) [13].The adverse effects of V on human health trigger the need for evaluating the mechanisms of vanadium toxicity in laboratory studies. As a result, many researchers examined the toxic and prooxidant effects of V on mammalian cells in cultures [14,15,16,17,18]. Among the reactive oxygen species (ROS) produced by V in cultured mammalian cells, hydrogen peroxide (H_2_O_2_) has been detected as the major ROS responsible for V-mediated cytotoxicity in many mammalian cell culture models [19,20,21]. Indeed, H_2_O_2_ is one of the most stable ROS, which due to its uncharged nature, can easily pass through cell membranes and induce toxic effects on cells in a direct or indirect manner [22,23].

However, a factor that was not taken into account in the above studies was the potential ability of V to react directly with the culture medium or probes used for oxidative stress testing. For example, in our previous work, we demonstrated the ability of V to induce H_2_O_2_ generation directly in DMEM/F12 culture medium, i.e., in a cell-independent manner [24]. Therefore, in this work, we decided to test the ability of vanadium to generate H_2_O_2_ also in other media used frequently for the cultivation of mammalian cells. In addition, we also tested the ability of V to react with the 2′,7′-dichlorodihydrofluorescein diacetate (DCFH-DA) and dihydrorhodamine 123 (DHR123) probes, which are very popular in the determination of V-induced ROS in mammalian cell cultures [14,16,19,25,26,27]. The tests were carried out in four media (DMEM/F12, DMEM, MEM, and RPMI), which as our analyses show differ in their pro-oxidative and antioxidant properties. Sodium metavanadate (NaVO_3_, [V(+5)]) and vanadyl sulphate (VOSO_4_, [V(+4)]) were tested in the present work since they are among the most common usable forms of V in in vitro research [17,19,20]. The study results contributed to the identification of potential artefacts that could be misinterpreted as oxidative stress induced by vanadium in cells in vitro.

## 2. Materials and Methods

### 2.1. Reagents

Dulbecco’s modified Eagle’s medium (DMEM) containing high glucose (25 mM), (Cat. No. 21063029), Dulbecco’s modified Eagle’s medium F-12 nutrient mixture (DMEM/F12) (Cat. No. 21041025), RPMI 1640 medium (Cat No. 11835030), and Minimum Essential Medium (MEM) (Cat No. 51200038) were all purchased from ThermoFisher Scientific. All the tested culture media were phenol free. Dihydrorhodamine 123, vanadyl sulphate hydrate (VOSO_4_·xH_2_O), sodium metavanadate (NaVO_3_), and foetal bovine serum (FBS) were purchased from Sigma Aldrich (St. Louis, MO, USA). The OxiSelectTM hydrogen Peroxide/Peroxidase assay and the OxiSelectTM Intracellular ROS assay (2′,7′-dichlorodihydrofluorescein diacetate) were provided by Cell Biolabs (San Diego, CA, USA). All reagents for the determination of antioxidant activity were purchased from Sigma Aldrich (St. Louis, MO, USA).

### 2.2. Preparation of NaVO_3_ and VOSO_4_ Stock Solutions

VOSO_4_·xH_2_O (assuming hydration of five molecules) was dissolved in deionised water to a final concentration of 10 mM stock solution (light blue colour). The stock solution was prepared freshly every time just before addition of VOSO_4_ to the tested culture media.

The stock solution of NaVO_3_ was prepared in deionised water at a 10 mM concentration and kept at 4 °C. The solution of NaVO_3_ used for the experiments was always colourless, indicating that it was a mixture of monomeric, dimeric, tetrameric, and pentameric vanadate [28].

### 2.3. Antioxidant Activity of Mammalian Cell Culture Media

#### 2.3.1. Ferric (III) Reducing Antioxidant Power (RP)

Reducing power was determined with the method developed by Pulido, Bravo, and Saura-Calixto [29]. Reducing power was expressed as Trolox equivalents (TE) in mg per 1 mL of medium.

#### 2.3.2. Ability to Quench ABTS Radicals (ABTS)

The experiments were carried out using the ABTS decolourisation assay [30]. The radical scavenging ability was expressed as Trolox equivalents (TE) in mg per 1 mL of medium.

#### 2.3.3. Ability to Quench Hydroxyl Radicals (OH)

The OH• scavenging ability was determined according to Su, Wang, and Liu [31]. It was expressed as Trolox equivalents (TE) in mg per 1 mL of medium.

#### 2.3.4. Ability to Quench DPPH Radicals (DPPH)

The experiments were carried out using the DPPH decolourisation assay [32]. The radical scavenging ability was expressed as Trolox equivalents in mg per 1 mL of medium.

### 2.4. Measurement of H_2_O_2_ Production in Cell Culture Media

The production of H_2_O_2_ in DMEM, DMEM/F12, RPMI 1640, and MEM without or with NaVO_3_ or VOSO_4_ was assessed using the OxiSelect Hydrogen Peroxide/Peroxidase Assay Kit. In this assay, the nonfluorescent probe ADHP (10-acetyl-3, 7-dihydroxyphenoxazine) is oxidised to the highly fluorescent resorufin in the presence of H_2_O_2_ and horseradish peroxidase (HRP).

For the assay, a 50 µL aliquot of each sample (appropriate culture medium with/without V compounds) was added into a black 96-well plate followed by 50 µL of the ADHP/HRP working solution to obtain a total volume of 100 µL. The fluorescence was measured 30 min after incubation at room temperature in a microplate reader (Synergy 2, BioTek Instruments, Inc., Winooski, VT, USA) at excitation/emission wavelengths of 540/590 nm. The concentration of H_2_O_2_ was derived from a standard curve obtained by adding different concentrations of H_2_O_2_ in assay buffer.

### 2.5. Oxidation of DCFH-DA and DHR123 Fluorogens in Cell Culture Media

The DCFH-DA and DHR123 assay protocol is based on the diffusion of DCFH-DA and DHR123 fluorogens into cells. Next, DCFH-DA (after deacetylation to non-fluorescent 2′,7′ dichlorodihydrofluorescein, DCFH) and DHR123 are oxidised by ROS to 2′,7′-dichlorofluorescein (DCF) and rhodamine 123 (RH-123), respectively [33].

For the experiments, the culture media (DMEM, DMEM/F12, RPMI 1640, and MEM) without or with NaVO_3_ or VOSO_4_ were added to black 96-well plates in a volume of 50 µL per well. Thereafter, 50 µL of the DCFH-DA or DHR123 dye solution was added to all wells (the final concentrations of DCFH-DA and DHR123 were 10 and 25 µM, respectively). The final concentrations of NaVO_3_ and VOSO_4_ in these wells were 100, 500, and 1000 µM. The fluorescence was measured after 15 min incubation at 37 °C in a humidified incubator using a microplate reader (Synergy 2, BioTek Instruments, Inc., USA) at excitation/emission wavelengths of 485/528 nm.

### 2.6. Data Analysis

Statistical analyses were performed using the Statistical Package for the Social Sciences (SPSS; SPSS Inc., IBM Corp., Armonk, NY, USA), version 25 (PS IMAGO PRO 5.1). All data are reported as means ± standard deviation. The significance of differences between the means was tested using one-way ANOVA with a post hoc Tukey test (equal variances) and Dunnett’s T3 test (unequal variances). All quantification results were obtained from at least two independent experiments, each of which consisted of at least three replicates per treatment to confirm data reproducibility and reliability.

## 3. Results and Discussion

### 3.1. Antioxidant Capacity of Mammalian Cell Culture Media

Table 1 shows the antioxidant activity of the mammalian cell culture media (DMEM, MEM, RPMI, and DMEM/F12), which was determined using established in vitro methods, i.e., DPPH radical scavenging, ABTS radical scavenging, and hydroxyl (OH) radical scavenging assays and a ferric (III) reducing antioxidant power (RP) assay. In general, these tests describe a different mode of antioxidant action [34]. The results obtained, especially from the ABTS test, generally agree with the results obtained for culture media by other authors [35]. At the same time, they confirm that the tested culture media have weak antioxidant capacity compared to serum (i.e., in vivo conditions) [35]. Halliwell [36] reported that the decreased level of antioxidants (compared to blood) is one of the main factors that further intensifies the prooxidative properties of cell culture media.

Among the media studied, the highest antiradical activity was determined for the DMEM medium, as shown by the DPPH, ABTS, OH, and RP tests, reaching values of 0.111, 0.844, 0.640, and 0.072 mg Trolox (TE)/mL, respectively. In turn, the lowest antioxidant capacity was found for MEM (DPPH test and OH assays) and RPMI (DPPH test and ABTS decolourisation assay); both these media exhibited the lowest antioxidant capacity in the RP test. The higher antioxidant activity of DMEM in comparison with the other cell culture media could be explained by the presence of the higher concentration of the antioxidant compounds. These include such amino acids as tyrosine, tryptophan, and methionine, which can significantly contribute to the antioxidant properties of mammalian cell culture media, as reported by Lewinska et al. (2007) [35]. In fact, as listed in Table 1, DMEM contains tyrosine (104 mg/L), tryptophan (16 mg/L), and methionine (30 mg/L) at a concentration approximately twice as high as in the other culture media tested.

Generally, among the bioassays used for the detection of antioxidant activity, the ABTS assay was the most sensitive in identifying the antioxidant capacity of culture media and showed the highest results (Table 1). This may be caused by the higher reactivity of the ABTS radical over other radicals (such as the DPPH radical) and the ability of the ABTS radical to react with compounds both through HAT (hydrogen atom transfer) and SET (single electron transfer) mechanisms [34]. In contrast, the RP assay demonstrated the lowest values of the antioxidant activity of the culture media. This may be associated with the fact that the antioxidant activity of some compounds (e.g., thiol-type antioxidants) which show low reactivity with ferric ions (Fe^3+^) and compounds which react with ferrous ions (Fe^2+^) cannot be accurately measured in this test [37,38].

### 3.2. Prooxidant Abilities of Mammalian Cell Culture Media

**Figure 1:**
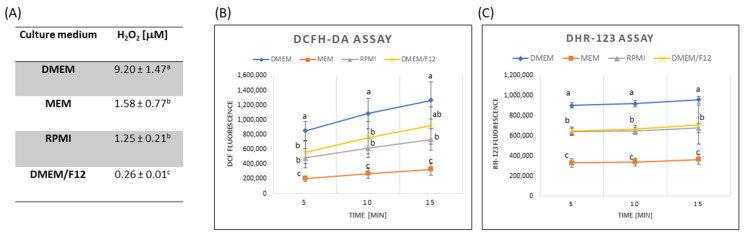
Reactive oxygen species (ROS) generation in mammalian cell culture media. (**A**) Production of H_2_O_2_ assessed by oxidation of acetyl dihydroxy phenoxazine (ADHP), (**B**) ROS production measured by oxidation of 2′,7′-dichlorodihydrofluorescein diacetate (DCFH_2_-DA), and (**C**) dihydrorhodamine (DHR-123). Cell culture media were incubated for 5 min (panel (**A**)) or 5–15 min (panels (**B**,**C**) at 37 °C, 5% CO_2_ atmosphere. The data represent the mean ± SD derived from 2 independent experiments each performed at least in four replicates. Means at the same time point followed by a common letter are not significantly different. The data were analysed by one-way ANOVA followed by Dunnett’s T3 post hoc test.

As shown in Figure 1A, the four media, i.e., DMEM, MEM, DMEM/F12, and RPMI, differ in the amount of generated H_2_O_2_ with the highest concentration of H_2_O_2_ (9.2 µM) in the DMEM medium and the lowest amount of H_2_O_2_ (0.26 µM) in the DMEM/F12 medium. The RPMI and MEM media showed intermediate values. The above data confirm that culture media can also be a source of some H_2_O_2_ formation. This may be caused by the common medium components such as HEPES and riboflavin, which were reported to be the main sources of light-induced H_2_O_2_ generation in the RPMI 1640 medium [39]. Among the four culture media tested in our study, the DMEM medium was the only one that had HEPES in its composition and the highest concentration of riboflavin (0.4 mg/L in DMEM compared with 0.100, 0.219, and 0.200 in MEM, DMEM/F12, and RPMI, Table S1). This may explain the highest H_2_O_2_ production among the media studied. On the other hand, the very low production of H_2_O_2_ in the DMEM/F12 medium can be explained, as this medium contains sodium pyruvate as a component. Sodium pyruvate is an optional ingredient of culture media serving as an additional and easily accessible energy substrate for cultivated cells. In addition, pyruvate undergoes rapid oxidative decarboxylation in a reaction with H_2_O_2_ yielding acetate, CO_2_, and water [40]. Thus, pyruvate leads to scavenging of H_2_O_2_ generated in culture media, which was probably responsible for the considerable differences in the H_2_O_2_ concentrations between DMEM/F12 and the other media tested in this study.

In addition, we assessed the ability of the media to oxidise DCFH-DA and dihydrorhodamine 123 fluorogen dyes. In agreement with other studies [41,42], it was found that the culture media significantly differed in their ability to oxidise these fluorogenic dyes (Figure 1B,C). We observed the highest DCFH-DA and DHR 123 oxidation rate in the DMEM medium (with the most complex composition) and the lowest in the MEM medium (the simplest composition). The increase in DCF fluorescence and RH-123 fluorescence in the RPMI and DMEM/F12 media exhibited intermediate values. The oxidation of these dyes may be partially related to the generation of ROS in culture media, as suggested by Martin-Romero et al. (2008) [42] and Grzelak et al. (2001) [41]. In fact, in our research, DMEM, which oxidised DCFH-DA and DHR123 most intensively, also had the highest levels of H_2_O_2_. Although H_2_O_2_ alone cannot oxidise DCFH or DHR123, the oxidation of these probes by H_2_O_2_ was noted in the presence of appropriate catalysts, such as free iron ions (present in the composition of DMEM and DMEM/F12) [43,44]. Moreover, other authors have shown that the oxidation of DCFH or DHR123 can also be stimulated by other redox active components of the medium, e.g., riboflavin [41] and tyrosine [45], which are present in the DMEM-type medium in the highest concentration, compared with other media. The high ability of the DMEM medium to oxidise these fluorescent probes may mistakenly obscure the level of oxidative stress induced by the tested substances.

### 3.3. NaVO_3_ and VOSO_4_ Modulate the Level of H_2_O_2_ in Mammalian Cell Culture Media

In this study, we elucidated the effects of NaVO_3_ and VOSO_4_ on the H_2_O_2_ level in the culture media due to the stability, mobility, and primary role of this ROS in V-mediated cellular damage [19,20]. As presented in Table 1, VOSO_4_ in all the concentrations tested (100, 500, and 1000 µM) significantly increased the H_2_O_2_ content in DMEM/F12 (from 4 to over 12-fold higher than in the control), which is in agreement with our previous observations [24]. We also observed that VOSO_4_ acted as a H_2_O_2_ stimulator in the MEM medium; however, in this medium, only the highest concentration of VOSO_4_ (1000 µM) produced nearly significantly (*p* = 0.051) higher amounts of H_2_O_2_ than in the control.

The induction of H_2_O_2_ by VOSO_4_ observed primarily in the DMEM/F12 medium may have been related to the oxidation of vanadyl by oxygen in a reaction described by Shankar and Ramasarma [46]. These authors suggested the following two-electron reduction of oxygen by vanadyl: 2 V(IV) + O_2_ + 2H+ → 2 V(V) + H_2_O_2_, but only in the presence of stimulating compounds exhibiting the oxygen radical quenching capability (e.g., citrate, histidine, ethanol, or mannitol). Among the culture media tested herein, DMEM/F12 has a complex formulation with respect to the presence of amino acids, vitamins, minerals, and other components (Table S1). Some of these substances are likely to act as stimulating compounds of the above-mentioned reaction. In accordance with our previous study, the VOSO_4_-induced H_2_O_2_ generation had a very low micromolar range (1–3 µM); hence, it cannot act as an artefact in V-induced effects on cells. In addition, as shown by us previously [24], the produced H_2_O_2_ almost completely disappeared within 1 h due to the presence of pyruvate, a strong H_2_O_2_ scavenger, in the DMEM/F12 formulation.

Interestingly, the tested V compounds exerted the opposite effect on the H_2_O_2_ concentration in DMEM, compared to DMEM/F12. In this medium, a significant decrease in the original H_2_O_2_ content was observed following the addition of NaVO_3_ or VOSO_4_ at all the concentrations tested. This indicates rapid decomposition of H_2_O_2_ by vanadium. The decomposition of H_2_O_2_ could be explained by the following Fenton-like reaction between vanadyl and H_2_O_2_: V(IV) + H_2_O_2_ → V(V) + OH^−^ + HO^•^ [47,48]. The ability to react with H_2_O_2_ in a Fenton-like reaction is not restricted only to vanadium, but this feature is also exhibited by some other transition metals such as copper (Cu^+^), silver (Ag), cerium (Ce^3+^), manganese (Mn^2+^) (reviewed by Hussain et al. [49]), and chromium (Cr^5+^) [50].

As presented in Table 2, the decrease in H_2_O_2_ content in DMEM was about 1 order of magnitude more intensive with VOSO_4_ compared to NaVO_3_. This might be due to the fact that vanadate from NaVO_3_ needs to be first reduced to vanadyl by medium components and the latter reacts with H_2_O_2_. In accordance, as evidenced very early by Keller et al. [51], the reaction between vanadate and H_2_O_2_ could not take place unless a superoxide anion was present which enabled reduction of vanadate to vanadyl, the latter being the reactive species that reacts with H_2_O_2_.

In order to better assess the effect of V on the level of H_2_O_2_ in the culture media, we conducted additional experiments with a medium enriched with 5% FBS (DMEM medium). The results obtained showed that the V-induced changes in H_2_O_2_ levels in the medium with 5% FBS are similar to those obtained in the medium without FBS (0% FBS) (Table S2). This result indicates that the presence of serum in the medium does not have a significant effect on the reactions induced by the tested V compounds in the culture medium. This might be due to the low serum capacity of binding this metal. Vanadium is present in blood serum mainly as a transferrin complex, whereas complexes with albumin occur at minor amounts [52]. In terms of the concentration, transferrin is less important in serum than albumin, which could be responsible for the lower buffering capacity of serum for V compared to other metals. In accordance with this prospect, studies by other authors showed that the cytotoxicity of V compounds against RLE-6TN cells was similar in DMEM/F12 medium without serum and with the addition of 5 and 10% serum [53].

### 3.4. NaVO_3_ and VOSO_4_ Significantly Increase the DCF and RH-123 Fluorescence in Mammalian Cell Culture Media (in the Absence of Cells)

**Figure 2:**
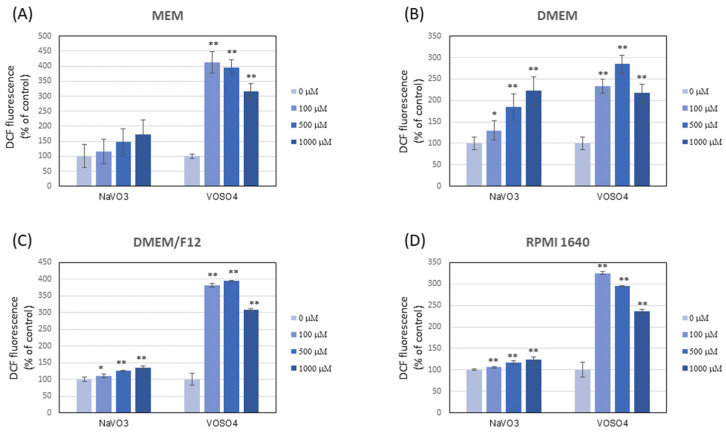
Oxidation of 2′,7′-dichlorodihydrofluorescein diacetate (DCFH-DA) to fluorescent dichlorofluorescein (DCF) in culture media after the addition of VOSO_4_ or NaVO_3_. The cell-free culture media were exposed to VOSO_4_ or NaVO_3_ (final concentrations of 100, 500, and 1000 µM) followed by the addition of the DCFH-DA probe (final concentration of 10 µM) and incubated for 15 min at 37 °C: (**A**) MEM; (**B**) DMEM; (**C**) DMEM/F12; (**D**) RPMI. The data are presented as means with error bars representing standard deviation. The data are representative of at least two independent experiments. Statistical differences between the control and the V-exposed media: * *p* < 0.05, ** *p* < 0.001 (one-way ANOVA followed by Dunnett’s T3 test).

**Figure 3:**
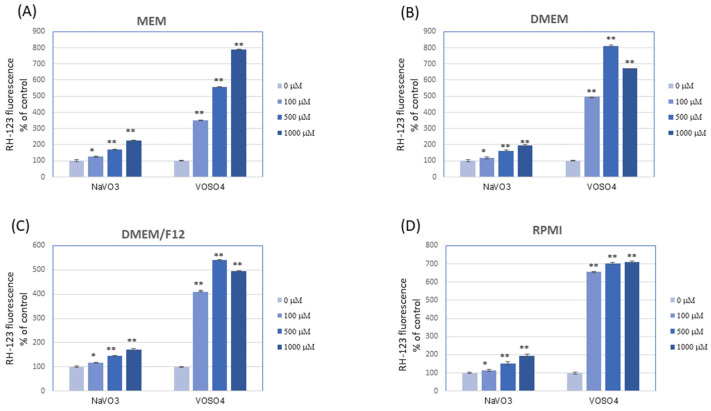
Oxidation of dihydrorhodamine 123 (DHR 123) to fluorescent rhodamine 123 (RH-123) in culture media after the addition of VOSO_4_ or NaVO_3_. The cell-free culture media were exposed to VOSO_4_ or NaVO_3_ (final concentrations of 100, 500, and 1000 µM) followed by the addition of the DHR 123 probe (final concentration of 25 µM) and incubated for 15 min at 37 °C; (**A**) MEM; (**B**) DMEM; (**C**) DMEM/F12; (**D**) RPMI. The data are presented as means with error bars representing standard deviation. The data are representative of at least two independent experiments. Statistical differences between the control and the V-exposed media: * *p* < 0.05, ** *p* < 0.001 (one-way ANOVA followed by Dunnett’s T3 test).

The results showed that NaVO_3_ and VOSO_4_ contributed to rapid oxidation of DCFH-DA and DHR 123 in all the tested culture media in cell-free conditions (Figure 2 and Figure 3). To our knowledge, this is the first work to show that vanadium is capable of interacting with DCFH-DA and DHR 123. Importantly, the type of medium generally did not affect the level of DCFH-DA and DHR 123 oxidation in the presence of respective V compounds, and the oxidation of both fluorogenic probes was much more intense following the VOSO_4_ addition versus NaVO_3_.

The reports important for this study indicate that the higher concentration of antioxidant components, e.g., pyruvate, tyrosine, or phenol red, in different cell culture media may significantly reduce the pro-oxidative properties of the tested substances [54,55]. However, in our research, despite the higher concentration of antioxidants in DMEM medium (and higher antioxidant activity) compared to other media, the pro-oxidative properties of respective V compounds against the tested DCFH-DA and DHR-123 fluorochromes in this medium were similar to other cell culture media (MEM, RPMI, and DMEM/F12). Thus, although the culture media used differed in their antioxidant (Table 1) and pro-oxidative properties (Figure 1), this ultimately had no effect on the interaction of vanadium with these dyes.

We suggest that vanadium can stimulate the oxidation of DCFH-DA and DHR 123 through direct interaction with these dyes or indirectly inducing redox reactions, e.g., with H_2_O_2_, the presence of which was confirmed in the media tested. So far, the results reported by other authors have shown that some transition metals (Fe^2+^ and Cu^+^) were able to oxidise DCFH [44,56,57], possibly through Fenton reactions, while others oxidized this dye very slowly (Mn^2+^) or not at all (Zn^2+^) [56]. Our research shows that, similar to Fe and Cu, vanadium can be classified as one of the metals capable of oxidising DCFH-DA (and DHR 123). In addition, as mentioned before, VOSO_4_ induced much more intensive oxidation of both probes in comparison with NaVO_3_. It can be speculated that additional reactions stimulated by vanadyl could be the reason. For example, reports show that during the interaction of vanadyl with H_2_O_2_, a peroxovanadate intermediate with good oxidizing properties was formed [58].

Higher reactivity of the DCFH-DA and DHR 123 assay in response to VOSO_4_ than NaVO_3_ may result in differences in the prooxidant effects between these two V compounds measured with these dyes on cultured cells. Accordingly, it has been reported previously that VOSO_4_ elicited higher ROS production in human lung carcinoma A549 cells [17] and osteoblast MC3T3E1 cells [59], compared with NaVO_3_, as assessed by DCFH-DA and DHR 123 oxidation, respectively.

## 4. Conclusions

This research showed that the studied mammalian cell culture media differ significantly in the antioxidant and pro-oxidative properties due to their different composition. Among the tested media, the DMEM medium, which contained the highest concentration of redox components (e.g., tyrosine, tryptophan, methionine, and riboflavin), showed the highest antioxidant abilities (measured by antioxidant bioassays) and pro-oxidative properties (measurement of the level of H_2_O_2_ and the oxidation of DCFH-DA and DHR 123 dyes).

Due to their redox active properties, the vanadium compounds, i.e., NaVO_3_ and VOSO_4_, were shown to modulate the level of H_2_O_2_ and to stimulate the oxidation of the DCFH-DA and DHR123 dyes in the tested culture media. The antioxidant and prooxidant abilities of the media tested had no influence on the effects of V on the H_2_O_2_ level and DCFH-DA and DHR 123 oxidation. Instead, the V compound type was of greater importance, with VOSO_4_ affecting the parameters tested much more intensively than NaVO_3_. The effect of V on the level of H_2_O_2_ in the culture media was in a range that should not affect the pro-oxidative properties of V towards cells. However, the interaction of V with DCFH-DA and DHR 123 observed in this research may artefactually interfere with the results of experiments investigating the effect of V on the generation of ROS obtained with these dyes in cell cultures, regardless of the type of culture medium in which the cells are grown.

## Figures and Tables

**Table 1:** Antioxidant properties of the cell culture media.

Medium	Antioxidant Activity			
	DPPH (mg TE/mL)	ABTS (mg TE/mL)	OH (mg TE/mL)	RP (mg TE/mL)
**DMEM**	0.111 ± 0.006 ^a^	0.844 ± 0.053 ^a^	0.640 ± 0.038 ^a^	0.072 ± 0.003 ^a^
**MEM**	0.025 ± 0.002 ^b^	0.492 ± 0.023 ^b^	0.150 ± 0.017 ^c^	0.013 ± 0.002 ^b^
**RPMI 1640**	0.029 ± 0.006 ^b^	0.319 ± 0.027 ^c^	0.321 ± 0.061 ^b^	0.013 ± 0.002 ^b^
**DMEM/F-12**	0.043 ± 0.009 ^c^	0.561 ± 0.031 ^d^	0.301 ± 0.054 ^b^	0.018 ± 0.004 ^c^

Means (± SD) in the columns followed by a common letter are not significantly different. The data were analysed by one-way ANOVA followed by Tukey post hoc test. DPPH—ability to quench DPPH radicals; OH—ability to quench OH radicals; RP—reducing power; ABTS—ability to quench ABTS radicals. Abbreviations: ABTS—2,2′-azino-bis(3-ethylbenzthiazoline-6-sulfonic acid, DPPH—di(phenyl)-(2,4,6-trinitrophenyl)iminoazanium, DMEM—Dulbecco’s modified Eagle’s medium, MEM—Minimum Essential Medium, DMEM/F-12—Dulbecco’s modified Eagle’s medium F-12 nutrient mixture, TE—Trolox equivalents.

**Table 2:** H_2_O_2_ in different cell culture media exposed to NaVO_3_ and VOSO_4_.

		[H_2_O_2_] µM at 5 min in			
Compounds Added (µM)		DMEM	MEM	RPMI	DMEM/F12
**none**		9.20 ± 1.47	1.58 ± 0.77	1.25 ± 0.21	0.26 ± 0.01
**VOSO_4_**	100	0.57 ± 0.41 ***	0.94 ± 0.15	0.87 ± 0.10 *	1.02 ± 0.14 ***
	500	0.77 ± 0.05 ***	2.62 ± 0.50	1.33 ± 0.04	2.28 ± 0.42 ***
	1000	1.15 ± 0.09 ***	4.11 ± 1.62	1.43 ± 0.02	3.25 ± 0.85 **
**NaVO_3_**	100	5.50 ± 1.14 ***	1.06 ± 0.47	0.73 ± 0.17 **	0.07 ± 0.01 *
	500	5.11 ± 1.10 ***	0.90 ± 0.40	0.60 ± 0.15 ***	0
	1000	3.98 ± 0.61 ***	0.68 ± 0.27	0.47 ± 0.11 ***	0

Note. VOSO_4_ or NaVO_3_ at final concentrations of 100, 500, and 1000 µM were added to the media and incubated at 37 °C for 5 min. H_2_O_2_ was then measured as described in the Material and Methods. The data are means ± SD, n ≥ 6. *** *p* < 0.001, ** *p* < 0.01, * *p* < 0.05 versus the respective medium control (one-way ANOVA followed by Dunnett’s T3 test).

## Data Availability

Not applicable.

## Supplementary Materials

The following supporting information can be downloaded at https://www.mdpi.com/article/10.3390/ijerph192215214/s1, Table S1: Composition of the culture media used for the experiments in this work, Table S2: H_2_O_2_ in DMEM with or without FBS supplementation exposed to VOSO_4_ or NaVO_3_
