# Supplementary material for: Identification of Potential Artefacts in In Vitro Measurement of Vanadium-Induced Reactive Oxygen Species (ROS) Production
Source: Int J Environ Res Public Health. 2022 Nov 18;19(22):15214. doi: 10.3390/ijerph192215214 (PMC9691132; doi:10.3390/ijerph192215214)
Supplement: Supplementary file 1 [file ijerph-19-15214-s001.zip › ijerph-1986488-supplementary.pdf]

Article

Identification of potential artefacts in *in vitro* measurement of vanadium-induced reactive oxygen species (ROS) production

Iwona Zwolak [1]*, Ewa Wnuk [2] and Michał Świeca [3]

1. Department of Biomedicine and Environmental Research, The John Paul II Catholic University of Lublin, Konstantynów Ave. 1J, 20-708 Lublin, Poland
2. Department of Biomedicine and Environmental Research, The John Paul II Catholic University of Lublin, Konstantynów Ave. 1J, 20-708 Lublin, Poland
3. Department of Biochemistry and Food Chemistry, University of Life Sciences, Skromna Str. 8, 20-704 Lublin, Poland

* Correspondence: iwona.zwolak@kul.pl; Tel.: +48-814-545-618 (I.Z.)

Table S1. Composition of the culture media used for the experiments in this work..

Components	MEM	DMEM	DMEM/F12	RPMI
**Aminoacids (mg l^{-1})**				
Gly	-	30	18.75	10
L-Ala	-	-	4.45	-
L-Arg	-	-	-	200
L-Arg · HCl	126	84	147.5	-
L-Asn	-	-	-	50
L-Asn· H_2O	-	-	7.5	-
L-Asp	-	-	6.65	20
L-Cys · HCl · H_2O	-	-	17.56	-
L-Cys · 2HCl	31	63	31.29	65
L-Glu	-	-	7.35	20
L-Gln	-	584	365	300
L-His	-	-	-	15
L-His· *HCl · H_2O*	42	42	31.48	-
L-Hyp	-	-	-	20
L-Ile	52	105	54.47	50
L-Leu	52	105	59.05	50
L-Lys · HCl	73	146	91.25	40
L-Met	15	30	17.24	15
L-Phe	32	66	35.48	15
L-Pro	-	-	17.25	20
L-Ser	-	42	26.25	30
L-Thr	48	95	53.45	20
L-Trp	10	16	9.02	5
L-Tyr · Na_2O_5	52	104	55.79	29
L-Val	46	94	52.85	20
**Vitamins (mg l^{-1})**				
Biotin	-	-	0.0035	0.2
Choline chloride	1	4	8.98	3

D-Calcium-pantothenate	1	4	2.24	0.25
Folic Acid	1	4	2.65	1
Niacinamide	1	4	2.02	1
Para-Aminobenzoic Acid	-	-	-	1
Pyridoxal · HCl	1	-	-	-
Pyridoxine · HCl	-	4	2	1
Riboflavin	0.1	0.4	0.219	0.2
Thiamine · HCl	1	4	2.17	1
Vit B12	-	-	0.68	0.005
i-Inositol	2	7.2	12.6	35
**Inorganic salts (mg l^{-1})**				
$CaCl_2$	200	200	116.6	-
$Ca(NO_3)_2 \cdot 4H_2O$	-	-	-	100
$CuSO_4 \cdot 5H_2O$	-	-	0.0013	-
$Fe(NO_3)_3 \cdot 9H_2O$	-	0.1	0.05	-
$FeSO_4 \cdot 7H_2O$	-	-	0.417	-
$MgCl_2$	-	-	28.64	-
$MgSO_4$	97.67	97.67	48.84	48.84
KCl	400	400	311.8	400
$NaHCO_3$	2200	3700	2438	2000
NaCl	6800	4750	6995.5	6000
$NaH_2PO_4 \cdot H_2O$	140	125	62.5	-
$Na2HPO_4$	-	-	71.02	800
$ZnSO_4 \cdot 7H_2O$	-	-	0.432	-
**Other components (mg l^{-1})**	-	-	-	-
D-Glucose (Dextrose)	1000	4500	3151	2000
HEPES	-	5958	-	-
GSH	-	-	-	1
Hypoxanthine Na	-	-	2.39	-
Linoleic Acid	-	-	0.042	-
Lipoic Acid	-	-	0.105	-
Putrescine ·2HCl	-	-	0.081	-
Sodium Pyruvate	-	-	55	-
Thymidine	-	-	0.365	-

MEM, Minimum Essential Medium; DMEM, Dulbecco’s modified Eagle’s medium; DMEM/F12, Dulbecco’s modified Eagle’s medium F-12 nutrient mixture

Table S2. H_2O_2 in DMEM with or without FBS supplementation exposed to $VOSO_4$ or $NaVO_3$

Compound added (μM)		$[H_2O_2]$ μM at 5 min in DMEM supplemented with	
		0% FBS	5% FBS
**None**		8.14 ± 0.13	8.67 ± 2.86
**$VOSO_4$**	100	0.42 ± 0.01***	1.83 ± 0.36**
	500	0.79 ± 0.01***	0.80 ± 0.05**
	1000	1.20 ± 0.01***	0.71 ± 0.12**
**$NaVO_3$**	100	4.43 ± 0.14***	4.16 ± 1.09*
	500	4.05 ± 0.05***	2.43 ± 0.49**
	1000	3.47 ± 0.01***	1.39 ± 0.20**

Note. $VOSO_4$ or $NaVO_3$ at a final concentrations of 100, 500 and 1000 μM were added to DMEM without FBS or with 5% FBS and incubated at 37°C for 5 minutes. H_2O_2 was then measured as described in Materials and Methods. Data are means ± SD, n=7. ***p<0.001, **p<0.01, *p<0.05 versus the respective medium control (one-way ANOVA followed by Dunnett's T3 test).
